# Intra-familial Transmission of Hepatitis B Virus Infection in Arak, Central Iran

**Published:** 2016

**Authors:** Masomeh Sofian, Mohammad Banifazl, Masoomeh Ziai, Arezoo Aghakhani, Ali-Asghar Farazi, Amitis Ramezani

**Affiliations:** 1 *Infectious Diseases Research Center (IDRC), Arak University of Medical Sciences, Arak, Iran*; 2 *Iranian Society for Support of Patients with Infectious Disease, Tehran, Iran*; 3 *Arak University of Medical Sciences, Arak, Iran*; 4 *Clinical Research Dept., Pasteur Institute of Iran, Tehran, Iran*

**Keywords:** Hepatitis B virus, Intra-familial, Transmission

## Abstract

**Background:**

The household transmission of hepatitis B virus (HBV) is a major health problem. High incidence of HBV infection is observed within the household contacts of HBV carriers. We aimed to evaluate serological markers of hepatitis B infection among family members of HBV carriers in Arak, central Iran.

**Methods:**

Data were collected from the 100 chronic HBV carriers (subjects with positive HBsAg for at least 6 months period) as index cases and 700 members of their family. Then, we checked serologic markers of hepatitis B [hepatitis B surface antigen (HBsAg), hepatitis B core antibody (anti-HBc) and hepatitis B surface antibody (anti- HBs)] using the ELISA test.

**Results:**

The prevalence rate of HBsAg, anti-HBs and anti-HBc among household members was 23.3%, 20.4% and 23% respectively. Isolated anti-HBc (positive anti-HBc with negative HBsAg and anti-HBs) found in 0.4% of family members. Mothers and children with 47.6% and 17.2% had the highest and lowest rates of HBV infection, respectively (*P*=0.00). There was a significant difference between mothers and spouses of index case (47.6% and 29.8%) regarding HBsAg positivity (*P*=0.03).

**Conclusion:**

The low rate of HBV infection reported in children reveal the effective prevention of HBV transmission with the universal vaccination programs and also importance of pregnant women screening for HBV serological markers.

## Introduction

Hepatitis B virus (HBV) infection is considered an important public health problem and has a high burden on health systems due to acute infection as well as its sequels like cancer and cirrhosis (1, 2). The household transmission of HBV is also a major health issue and raise concern for patients and their family as well as health system (3). High rate of HBV infection seen in the household contacts of chronic HBV carriers as the prevalence of HBV transmission within carriers’ family members reaches to 11- 57%. (4-6). “The risk of transmission is variable based on socio-demographic situation, family role (as infected mothers increase the risk of transmission) and viral markers of the index case” (6-8).

The major routes of HBV transmission include blood, sexual contacts, vertical (from infected mothers to offspring) and horizontal (between the spouses or children) transmission (5). In regions where the HBV infection is endemic, vertical transmission and horizontal transmission during early childhood have a remarkable role in transmission of HBV among family members. In low HBV endemic regions like North America and Western Europe, the major route of transmission is through sexual contacts and injecting drug use (9, 10). In these areas, vertical transmission of HBV is a rare event.

Since 1993, a nationwide HBV vaccination program was launched in Iran. This program resulted in a significant reduction in the rate of persistent HBV infection, hepatocellular carcinoma and fulminant hepatitis in Iran (11, 12). Now Iran is an intermediate to low endemic area for HBV infection and it is estimated that in the near future HBV elimination will be possible (11). Additionally, the risk factors for acquiring HBV infection have changed from vertical or early childhood to adolescents (11). 

Although intra-familial transmission of HBV was evaluated in several regions but investigation in different areas, can provide valuable information about the routes of HBV transmission in population and help in discovering the predominant mode of intra-familial HBV spread and local features. 

Therefore, we aimed to evaluate serological markers of hepatitis B infection among family members of chronic HBV carriers in Arak, Iran. 

## Materials and Methods

**Study population**

In this cross-sectional study, 100 chronic HBV carriers (subjects with positive HBsAg for at least 6 months period) as index cases and 700 members of their family enrolled from May 2012 to February of 2013 in Arak, central Iran. All the subjects were recruited by trained interviewers and data were collected by a validated questionnaire. Questionnaires were completed by a physician and sampling was done by a trained person. 

This project was approved by Arak University of Medical Sciences Ethical Committee and informed consent was obtained from subjects prior to their enrollment.

**Serological assessment**

The serum HBsAg, anti-HBs and anti-HBc levels were tested by ELISA. The commercial enzyme immunoassay kits used were Dia.Pro Diagnostic BioProbes, Milan, Italy. Serum samples with an anti-HBs level ≥10 IU/L were considered as protective against HBV infection. 

**Statistical analysis**

Statistical analyses were conducted using SPSS (version 16, Chicago, IL, USA). The Chi square was used to compare variables. Data are presented as mean ± SD or, when indicated, as an absolute number and percentage. *P*-values <0.05 were considered statistically significant.

## Results

 A total of 100 (62% male: 38% female) chronic HBV carriers (mean age: 38.09 ±9.7 yr) and 700 (51.7% male: 48.3% female) members of their family (mean age: 42.02 ±14.7) were enrolled in the study. The family members include 82 mothers, 78 fathers, 57 spouses, 192 children, and 291 siblings. The distribution of hepatitis B virologic markers in family members of index cases is shown in Table 1.

**Table 1:** Frequency distribution of hepatitis B markers in family members of index cases, n (%)

Family Member	HBsAg Positive	Anti-HBs Positive	Anti-HBc Positive	Isolated anti-HBc
**Father (n=78)**	14(17.9)	19(24.4)	19(24.4)	0(0)
**Mother (n=82)**	39(47.6)	12(14.6)	47(57.3)	0(0)
**Wife (n= 28)**	11(39.3)	2(7.1)	3(10.7)	0(0)
**Husband (n=29)**	6(20.7)	6(20.7)	6(20.7)	0(0)
**Brother (n=165)**	35(21.2)	31(18.8)	20(12.1)	0(0)
**Sister (n=126)**	25(19.8)	25(19.8)	20(15.9)	3(2.4)
**Son (n=91)**	13(14.3)	28(30.8)	18(19.8)	0(0)
**Daughter (n=101)**	20(19.8)	20(19.8)	28(27.7)	0(0)

Overall among the family members were evaluated, 23.3% were positive for HBsAg. The HBsAg positivity rate was determined in the family members with respect to their relationship to the index cases. Mothers and spouses with 47.6% and 29.8%, respectively, had the highest rates of HBsAg positivity in the family members and the lowest rate was related to siblings (20.6%) and children (17.2%).

The overall prevalence of anti-HBc positivity among family members was 23%. Anti-HBc was positive in 15.8% of spouses, 13.7% of the siblings and 23.9% of children of index cases. The overall prevalence of anti-HBs among family members was 20.4%. Anti-HBs were positive in 14% of spouses, 19.2% of the siblings, and 24% of children of index cases.

The prevalence rate of isolated anti-HBc (positive anti-HBc with negative HBsAg and anti-HBs) among household members was 0.4%. 

Mothers and children with 47.6% and 17.2% had the highest and lowest rates of HBV infection, respectively (*P*=.000). There was a significant difference between mothers and spouses of index case (47.6% and 29.8%) regarding HBsAg positivity (*P*=0.03). The prevalence of HBV infection was 39.3% in women with infected husbands and 20.7% in men with infected wives (*P*=0.12).

## Discussion

This survey investigated serological markers of hepatitis B infection among family members of chronic HBV carriers in Arak, Iran. The prevalence rate of HBsAg, anti-HBs and anti-HBc among household members was 23.3%, 20.4% and 23% respectively. Moreover, mothers and children with 47.6% and 17.2% had the highest and lowest rates of HBV infection, respectively. 

HBV may be transmitted through parenteral, sexual, vertical and horizontal contacts (6). Household members of HBV positive carriers are at increased risk of infection (13, 16) and clustering of HBV infection within the family members is common (17). Spouses were frequently being anti-HBs positive while siblings or the parents were often being HBsAg positive. In addition, infected mothers are the main reservoirs of infection leading to vertical and horizontal transmission (18). HBsAg positivity in mothers of index cases was more frequently than that in their spouses. Therefore, mother to child HBV transmission is more efficient way for transmission of HBV than sexual route (18). 

After implementation of universal vaccination program in Iran since 1993, the rate of new infection is reduced (12) and the risk factors of acquiring HBV infection have changed from vertical or early childhood to adolescents (19, 20). In the present study, the high rate of HBV infection in mother of index cases confirmed that the vertical transmission is a main route of HBV transmission and more efficient than sexual transmission. Moreover, the low rate of infection in children could be attributed to the effect of national vaccination program and screening of pregnant women for serological markers of HBV in Iran. Therefore, disruption in the chain of mother to infant transmission by continuing the universal vaccination program could remove this main route of transmission.

Previous studies in different parts of Iran had demonstrated a variety of seroprevalence rates ranging from 6% in Golestan Province (21) to 10.6% in Guilan Province (22), 11% in Nahavand (6), 22.2% in Babol (23) and 37.1% in Hamadan cities (24).This seroprevalence rate in other countries was from 12.1% in Bosnia and Herzegovina (19), 12.2% in Egypt (4), 14.1% in South Korea (25),16% in Australia (26),18.8% in Greece (13), 19.4% in India (15), 21.1% in Brazil (5),30.5% in Turkey (8) and 33.5% in Spain (27). In the present study, 23.4% of the family members were infected with HBV, which is in accordance to Roushan et al. a result (23).

Isolated anti-HBc is defined as positive anti-HBc with negative HBsAg. This rate was variable in different studies on family members in Iran as 1.3% in Isfahan (28), 2.5% in Nahavand (6), and 3.3% in Zahedan (18). Isolated anti-HBc reported in 0.4% of household members in our cohort of study, which is lower than other surveys, carried out in family members from Iran (6, 18, 28). Isolated anti-HBc is most frequently associated to occult HBV infection (OBI) raised some concern due to harbors risk of HBV transmission and association with hepatocellular carcinoma (29). Therefore, low rate of isolated anti-HBc in our study can reflect the low frequency of OBI circulation in Intra-familial exposure.

## Conclusion

 This study evaluated the serological markers of hepatitis B infection among family members of chronic HBV carriers in Arak, Iran. The low rate of HBV infection reported in children in this study reveal the effective prevention of HBV transmission with the universal vaccination programs and importance of screening of pregnant women for HBV serological markers. 
